# Dual-mode hyperbolicity, supercanalization, and leakage in self-complementary metasurfaces

**DOI:** 10.1515/nanoph-2023-0076

**Published:** 2023-05-08

**Authors:** Enrica Martini, Federico Giusti, Alice Benini, Stefano Maci

**Affiliations:** Department of Information Engineering and Mathematics, University of Siena, Siena, Italy

**Keywords:** antennas, Babinet’s principle, leaky-waves, metasurfaces, phased array, self-complementarity

## Abstract

Anisotropic Self-Complementary Metasurfaces (SC-MTSs) are structures constituted by an alternation of complementary inductive and capacitive strips, which are “self-dual” according to Babinet’s duality principle. They support the propagation of two orthogonally polarized surface-wave modes with the same phase velocity along the principal directions (i.e., along the strips and normal to them). The isofrequency dispersion curves of these modes are hyperbolas, and therefore, these MTSs fall in the category of hyperbolic MTSs. It is shown here that the hyperbolic dispersion curves may degenerate in same cases into almost straight lines, which implies that the velocity of energy transport is constantly directed along the same direction for any possible phasing orthogonal to the strips. In this circumstance, the SC-MTS can be conveniently used to design dual-polarized leaky-wave antennas by modulating the impedances of the complementary strips.

## 1 Introduction

Metasurfaces (MTSs) [1–4] are thin layers of subwavelength elements employed to control wavefronts of guided waves [5–10], scattered waves (SWs) [11–14] and leaky waves (LWs) [15–26]. Due to their reduced thickness and sub-wavelength patterning, they can conveniently be described in terms of equivalent boundary conditions (BCs) of impedance type.

**Figure 1:**
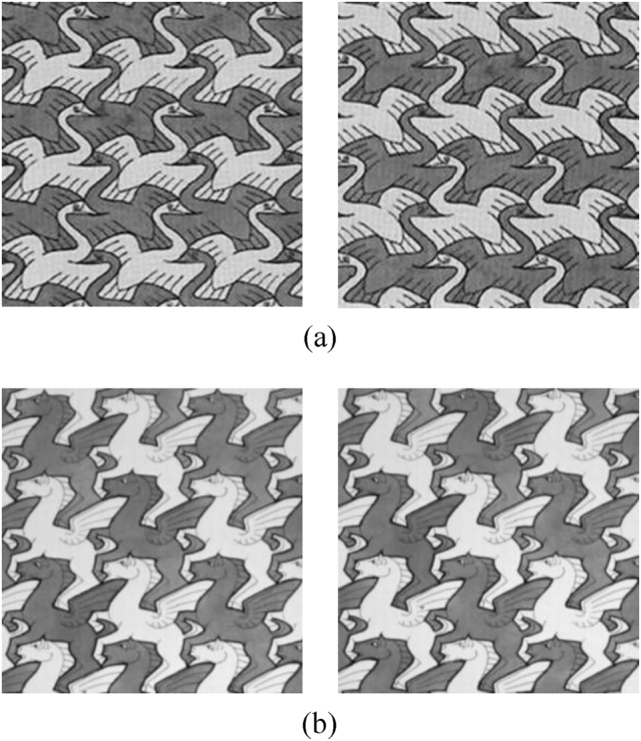
Details of Mauritius Cornelis Escher’s pieces; (a) black and white swans; (b) black and white Pegasuses. Swapping black and white implies a shift of the image in the vertical or the horizontal direction and the inversion of the direction of the swans (not the one of the Pegasuses).

An interesting class of MTSs is represented by MTSs exhibiting self-complementary properties (SC-MTS), i.e., with a fundamental periodic cell which is invariant under complementary inversion (i.e., in a single-layer metallic structure, interchanging the metal with free space) except for a translation and/or rotation in the MTS plane. These MTSs are characterized by a self-complementary patterning, and their layout, therefore, resembles some pictures made by the famous artist M.C. Escher. Examples of these latter are shown in Figure 1. There, black and white self-complementary pictures of animals intersect each other so that swapping the white with the black shifts the picture of half a period and possibly switches the direction of the animals (Figure 1(a)).

Another interesting class of MTSs is constituted by the so-called Hyperbolic MTSs, that exhibit hyperbolic type iso-frequency dispersion curves for SWs [27–29]. Many regimes of hyperbolic SW propagation were discovered and analysed, including negative refraction [30], zero-index [31], Moiré patterns [32, 33], unidirectional propagation [34, 35] and canalization [36]. In [37], SC-MTSs have been demonstrated to provide hyperbolicity and extreme canalization with TE-TM modes degeneracy. Furthermore, it has been shown theoretically and experimentally that the hyperbolic regime for SC-MTSs can be realized at all frequencies by using the duality conditions.

In this paper, we further investigate this class of MTSs, starting from single layer structures and then extending the analysis to the case of two coupled metalayers. The presentation of the paper is structured as follows. Section 2 discusses the concept of self-complementarity and self-duality for single layer MTSs. Section 3 presents the SW phenomenology in SC-MTSs with special emphasis on the hyperbolic behaviour. Section 4 shows how with a two-layer structure it is possible to obtain straight isofrequency dispersion curves, and Section 5 illustrates how this kind of structures can be conveniently used to design dual-polarized leaky wave antennas. Finally, Section 6 draws some conclusions.

## 2 Self-complementarity and self-duality

**Figure 2:**
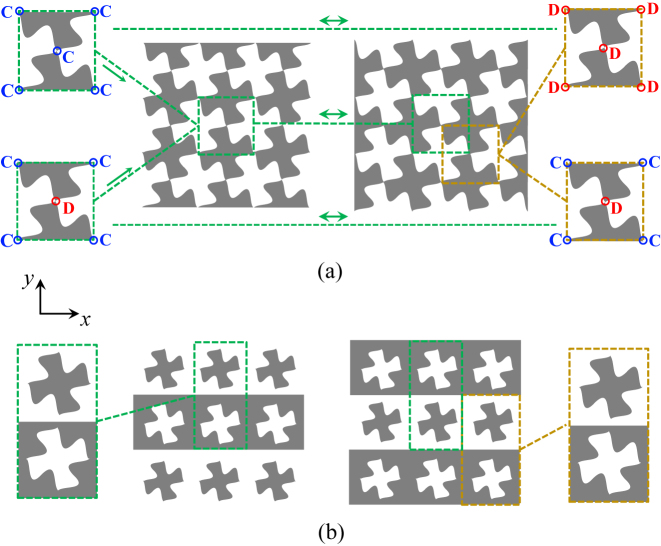
Self-complementary metasurfaces; (a) checkerboard-like MTS with two different types of vertex connections. Top insets: isotropic MTS with all the vertexes connected. Bottom insets: anisotropic MTS with vertexes alternatively connected and disconnected along the *x*-direction. (b) Anisotropic MTS consisiting of metal strips periodically etched with slots alternating with equal width free space regions where complementary metallic patches are realized.

In order to investigate the interaction between an electromagnetic wave and an SC-MTS it is convenient to refer to the basic class of SC-MTSs consisting of a single-layer periodically patterned metal floating in free-space. A rectangular reference system (*x*, *y*, *z*) is defined with the *z* axis normal to the surface. Different behaviors can arise depending on the degree of symmetry of the unit cell. Two of them, particularly interesting for our purposes, are presented in Figure 2.

In Figure 2(a), the unit cell rotates by 90° under complementary inversion (or, equivalently, it shifts of half a period in both the principal directions). A checkerboard MTS is a particular case of this kind of structures [38–41]. Under homogenization assumption, at low frequency, SC-MTSs of this type are almost isotropic; that is, they exhibit impedance BCs almost independent of the wave propagation direction. These SC-MTSs do not satisfy self-duality, namely the Babinet’s self-complementarity of the fields. The reason for this is related to the fact that in this kind of structures there must be some vertex points where the edges of the metalizations merge. At these points, the material is not univocally defined, and the attribution of metal or free-space conditions drastically changes the nature of the MTS. In fact, when the vertex points are assumed to be metal (upper left inset in Figure 2(a)), the behaviour of the MTS at low frequency is inductive, since it admits a flow of electric current in any possible direction. This implies that the MTS in Figure 2(a) supports a surface wave (SW) of transverse magnetic (TM) type with respect to the normal axis without cut-off. Its complementary inversion leads to the configuration shown in the upper right inset of Figure 2(a), in which all the vertex points are disconnected. In this case, the low frequency behaviour is capacitive, and therefore the MTS supports a transverse electric (TE) SW without cut-off. The two modes can be demonstrated to have the same phase velocity due to the Babinet’s properties [42]. It is clear, however, that TE and TM waves cannot be supported simultaneously on the structure, since they exist only for different status of the vertexes (namely, the MTS is not self-dual). On the other hand, the vertex points may be used to control the low-frequency transmission properties of the MTS by electronic [38–40] or optical [41] switching. This may also serve to create reconfigurable propagation paths for SWs propagating on the surface [42].

On the contrary, the same SC-MTS of Figure 2(a) is also *self-dual* if the central point of the unit cell is disconnected and the boundary points are connected, as in the bottom left inset. In fact, in that case the complementary inversion of the geometry provides a structure characterized by the same unit cell, with just a shift of half a period in the two principal directions (see bottom right inset in Figure 2(a)). The resulting MTS is strongly anisotropic, and it can support two orthogonal SW modes with the same phase velocity at any frequency along the direction where the elements are connected (the horizontal one, in Figure 2(a)).

Another example of MTS that intrinsically respects the Babinet’s principle is shown in Figure 2(b), where metal strips periodically etched with slots alternate with equal width free space regions where complementary metallic patches are realized. In this case, the unit cell rotates by 180° under complementary inversion (or, equivalently, it shifts of half a period in the vertical direction). Under homogenization assumption, the structure behaves similarly to the one in the bottom of Figure 2(a). However, in this case there is no ambiguity in the definition of the complementary structure. Hence, this SC-MTS is self-dual, namely, it supports the simultaneous propagation along the strips of two degenerate orthogonal modes.

The type of SC-MTS in Figure 2(b) has been used to design frequency-selective filters [43], perfect absorbers [44] and linear-to-circular polarization converters for incident plane waves [45, 46]; furthermore, it has been shown to have frequency-constant transmission properties when excited by circularly-polarized waves [47].

## 3 SW phenomena in SC-MTSs

**Figure 3:**
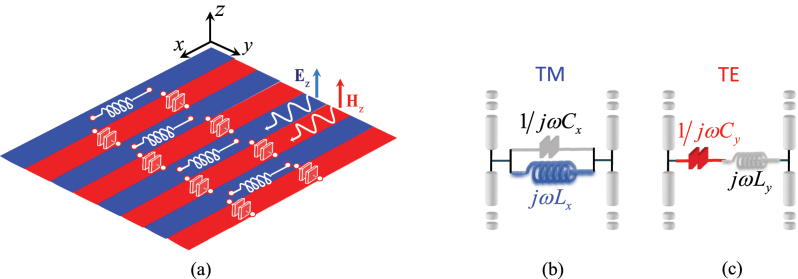
Equivalent circuit models for self-complementary MTS constituted by narrow strips of inductive (blue) and capacitive (red) reactances floating in free-space. (a) Lumped components denote the low frequency behaviour of the surface impedance along *x* (inductance) and along *y* (capacitance). (b) and (c): *z*-transmission lines for dispersion analysis of TM (b) and TE (c) SWs propagating along *x*.

In this section, we study the dispersion properties of surface waves supported by SC-MTSs of the type illustrated in the bottom of Figure 2(a) and in Figure 2(b). We assume the lattice period to be small in terms of a wavelength, so that the boundary conditions can be homogenized along the strips. Under this condition, the structure can be modelled as an infinite alternation of complementary inductive and capacitive impedance strips (see Figure 3).

### 3.1 Dual-mode hyperbolic isofrequency dispersion curves

To analyse the dispersion behaviour of the SWs supported by the anisotropic SC-MTS, we exploit the *z*-transmission line analogue of the MTS, as shown in Figure 3(b) and (c). We observe that, for symmetry reasons, the transmission lines associated with TM and TE modes are rigorously decoupled for propagation along *x* and *y*, while they are in general coupled for other directions of propagation. Along *x*, the structure supports both a TM and a TE mode without cut-off and with the same phase velocity. The propagation constant of these modes is found solving the resonance equation of the two circuits in Figure 3(b) and (c), respectively, where the equivalent impedances of the shunt loads satisfy the Babinet’s principle [47], namely *X*_*y*_ *X*_*x*_ = −*ζ* ^2^/4, where $\zeta =\sqrt{\mu_{0}/\epsilon_{0}}$ is the free space impedance and *ϵ*_0_ and *μ*_0_ are the free space permittivity and permeability, respectively. Imposing *X*_*y*_ *X*_*x*_ = −*ζ* ^2^/4 at the resonance frequency and at low frequency, one obtains $1/\sqrt{L_{y}C_{y}}=1/\sqrt{L_{x}C_{x}}$ and *L*_*x*_/*C*_*y*_ = *ζ* ^2^/4. Therefore, when the strips are narrow in terms of a wavelength (*kd* ≪ *π*, where *k* is the free space wavenumber and *d* is the lattice period of the unit cell), the MTS can be homogenized through the following boundary conditions

(1) $$E_{t}\left(z=0\right)=j\underline{\underline{X}}\cdot \hat{z}\times \left[H_{t}\left(z=0^{+}\right)-H_{t}\left(z=0^{-}\right)\right]\;$$

(2) $$\underline{\underline{X}}=\frac{\zeta }{2}\left(\eta_{x}\hat{x}\hat{x}+\eta_{y}\hat{y}\hat{y}\right)$$

(3) $$\eta_{x}=-\eta_{y}^{-1}=\frac{\omega /\omega_{B}}{\left(1-\omega^{2}/\omega_{0}^{2}\right)}$$

where *η*_*x*_ = 2*X*_*x*_/*ζ*, $\omega_{B}≐\zeta /\left(2L_{x}\right)=2/\left(C_{y}\zeta \right)=1/\sqrt{L_{x}C_{y}}$ and $\omega_{0}≐1/\sqrt{L_{y}C_{y}}=1/\sqrt{L_{x}C_{x}}$ . In (1), **E**_*t*_ and **H**_*t*_ are the tangential components of the electric and magnetic fields at the MTS, while $j\underline{\underline{X}}$ is the effective surface impedance tensor describing the anisotropic MTS. Equations (1)–(3) and the consequent dispersion equations apply to both the structure in the bottom of Figure 2(a) and the structure in Figure 2(b).

Along a generic propagation direction, the structure supports two different modes, one quasi-TM (*q*-TM) and one quasi-TE (*q*-TE), with different phase velocities. Following the procedure described in [48], one can find the dispersion equation for an arbitrary direction of propagation ${\hat{k}}_{t}=\cos\;\alpha \hat{x}+\sin\;\alpha \hat{y}$ , which leads to the so called “isofrequency dispersion curves” (IDCs). At a fixed angular frequency *ω*, these curves are the locus described by the end-point of the transverse wave vector $k_{\text{SW}}=\beta_{\text{SW}}\left(\alpha \right){\hat{k}}_{t}$ , i.e., they show the transverse wavenumber as a function of the propagation angle $\alpha =\arccos\left({\hat{k}}_{t}\cdot \hat{x}\right)=\arccos\left(k_{x}/k\right)$ . In other terms, each IDC represents the cut at a given angular frequency *ω* of the dispersion surface *ω* = *ω*(**k**_SW_). Since the supported modes are SWs, these curves are always outside the circle of radius *k*.

While the general expression of IDCs for anisotropic metasurfaces is given in [48], an explicit expression for the SC-MTSs considered in this paper was derived in [37]. For positive *k*_*x*_ and *ω* < *ω*_0_ these IDCs are written as

(4) $$\left(k_{x}+\eta_{x}|k_{y}|-k\sqrt{1+\eta_{x}^{2}}\right)\left(k_{x}-\eta_{x}|k_{y}|-k\sqrt{1+\eta_{x}^{2}}\right)=0$$

where the vanishing of the first factor is associated with the *q*-TM mode and the vanishing of the second one is associated with the *q*-TE mode. A solution for the two IDCs exists in the following regions of the *k*-space

(5) $$\frac{k}{\sqrt{1+\eta_{x}^{2}}}\leq k_{x}\leq k\sqrt{1+\eta_{x}^{2}}\;\left(q-TM\right)$$

(6) $$k_{x}\geq k\sqrt{1+\eta_{x}^{2}}\;\left(q-TE\right).$$

For negative *k*_*x*_, the dispersion equation can be obtained by symmetry with respect to the *k*_*y*_ axis.

**Figure 4:**
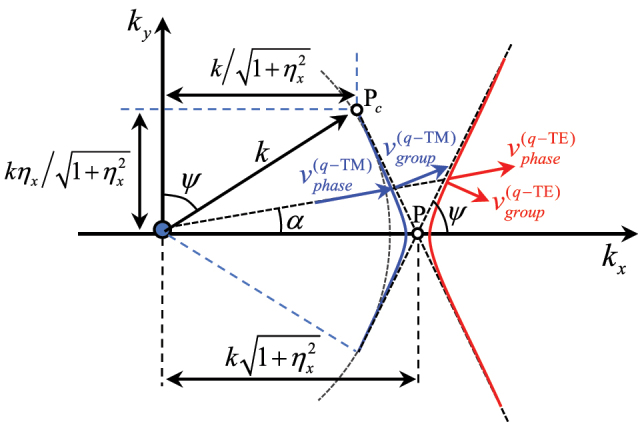
Detail of the IDCs at the crossing point of TE and TM modes for SC-MTSs. If small losses are introduced, the straight lines are split in two almost touching hyperbolas. Group and phase velocities are also depicted. The group velocities are orthogonal to the IDCs and parallel to the real part of the Poynting vectors of the SWs; these group velocities unit vectors are opposite in sign along the *y*-direction.

Each of the two IDCs solution of (4) consists of two straight lines branches with the cusp at the spectral point $\left(k_{x},k_{y}\right)=\left(k\sqrt{1+\eta_{x}^{2}},0\right)\equiv P$ . At P, the two modes become purely TM and TE, respectively, and their propagation constants degenerate into $\beta_{SW}=k\sqrt{1+\eta_{x}^{2}}$ . However, in presence of small losses, the dispersion curves become hyperbolas (Figure 4). This effect can be modelled by equating the right hand side of (4) to a vanishingly small positive number. The dual hyperbolic behaviour also arises in practice in lossless structures due to the fact that the individual strips are not perfectly described by local isotropic boundary conditions; this is due to a different interaction along *x* and *y* for elements of the same nature (slots or patches). However, this has a small impact on the overall phenomenology.

We note that for a certain angle *α* the IDC of the *q*-TM mode is tangent to the circle of radius *k*, which is the boundary of the visible region. This means that when the phase shift in direction orthogonal to the strips is increased, the *q*-TM mode encounters a cutoff; this happens at the spectral point $\left(k_{x},k_{y}\right)=\left(k/\sqrt{1+\eta_{x}^{2}},k\eta_{x}/\sqrt{1+\eta_{x}^{2}}\right)\equiv P_{c}$ . When the MTS is printed on a dielectric slab, the *q*-TM IDCs are tangential to the circle of radius equal to an effective wavenumber *k*_eff_ > *k*. For a generic propagation direction, the vectorial phase-velocity $v_{\text{phase}}^{\left(q-\text{TM},q-\text{TE}\right)}=\left[\omega /\beta_{\text{SW}}^{\left(q-\text{TM},q-\text{TE}\right)}\right]{\hat{k}}_{t}$ and the group-velocity $v_{\text{group}}^{\left(q-\text{TM},q-\text{TE}\right)}=\nabla_{\beta_{\text{SW}},\alpha }\omega$ are not aligned each other, as it usually happens for strongly anisotropic MTSs, and the group velocity is different for the two modes. The group velocity is directed along the normal to the IDCs and coincides with the velocity of energy transport [10, 48]. In particular, it is seen that the *y*-component of the group velocity unit vector has the same amplitude and opposite sign along the *y*-direction for *q*-TE and *q*-TM modes (Figure 4).

The asymptotes of the two hyperbolas associated with the *q*-TE and *q*-TM modes form an angle Ψ and (*π* − Ψ) with the *x*-axis, respectively, where

(7) $$\Psi =\arccos\left(\frac{\eta_{x}}{\sqrt{1+\eta_{x}^{2}}}\right).$$

It is seen that the angle Ψ approaches *π*/2 for *η*_*x*_ ≪ 1, which occurs for

(8) $$\omega \ll \omega_{B}=1/\sqrt{L_{x}C_{y}}$$

(9) $$\omega <\omega_{0}=1/\sqrt{L_{x}C_{x}}=1/\sqrt{L_{y}C_{y}}$$

the second inequality being implicit in the solution (4).

**Figure 5:**
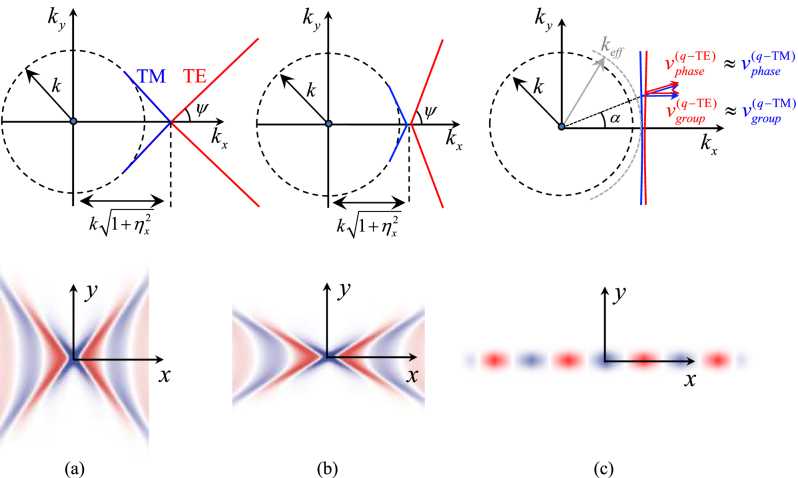
Evolution of the IDCs for different values of *ω*_*B*_ at the same frequency (top) and corresponding surface field patterns when a vertical magnetic dipole placed close to the surface excites a TE SW (bottom). (a) Low value of *ω*_*B*_/*ω*_0_ and (b) high value of *ω*_*B*_/*ω*_0_. For increasing value of *ω*_*B*_/*ω*_0_ (decreasing value of the product *L*_*x*_ *C*_*y*_) the IDCs slope increases and finally the two lines become almost parallel to the vertical axis. (c) Degeneration of the IDCs into straight lines tangent to the circle of radius equal to the effective *k*. In this case, *ω*_*B*_/*ω*_0_ → ∞ and the SW field is concentrated along the direction of the strips.

**Figure 6:**
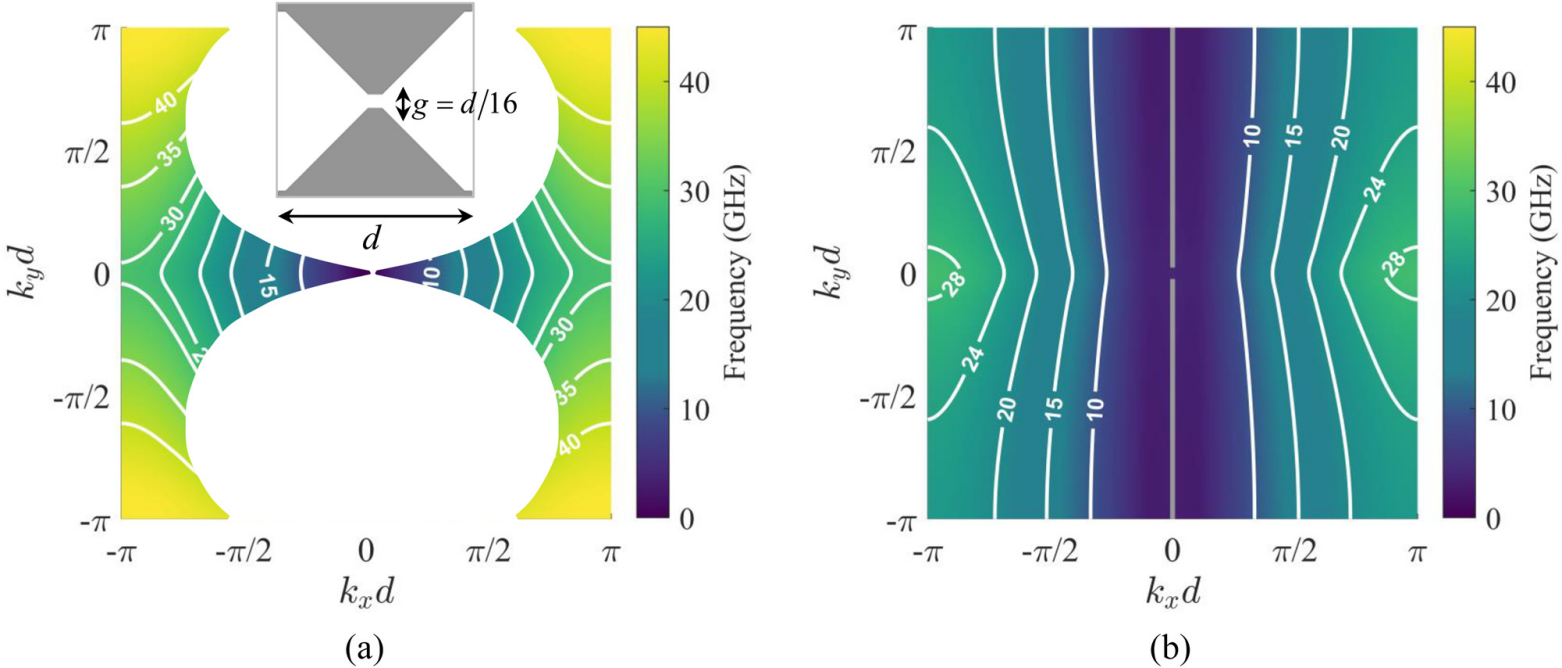
IDCs for a checkerboard SC-MTS with connected vertexes along the *x*-direction. The lattice period is *d* = 4 mm. (a) TM mode. (b) TE mode. The frequencies are represented by a color map, and the white isolines represent the IDC for a given frequency (indicated in GHz in the label). In the TM case, the white region indicates phase combinations for which SW propagation is prevented; its boundary is formed by the points of contact between the IDCs and the circular light line for the different frequencies.

As the ratio *ω*_*B*_/*ω*_0_ increases for a given frequency (that is, a given electrical dimension of the periodic cell), the slope of the asymptotes becomes steeper for larger ratios. Eventually, when this ratio becomes very large, the asymptotes tend toward vertical lines. However, in this limit, the *q*-TM remains confined in a very small angular region, and for increasing values of *α* it becomes a purely TEM wave with *z*-directed E-field (the IDC touches the visible circle); we note indeed that this TEM wave is compliant with the boundary conditions, irrespective of the metalization shape. This evolution is illustrated in Figure 5(a) and (b). Figure 5(c) represents a solution with vertical IDCs, that will be discussed in Section 4.2. An example of full wave analysis, relevant to a checkerboard MTS with a small gap, is reported in Figure 6. The results are obtained using CST Microwave Studio.

**Figure 7:**
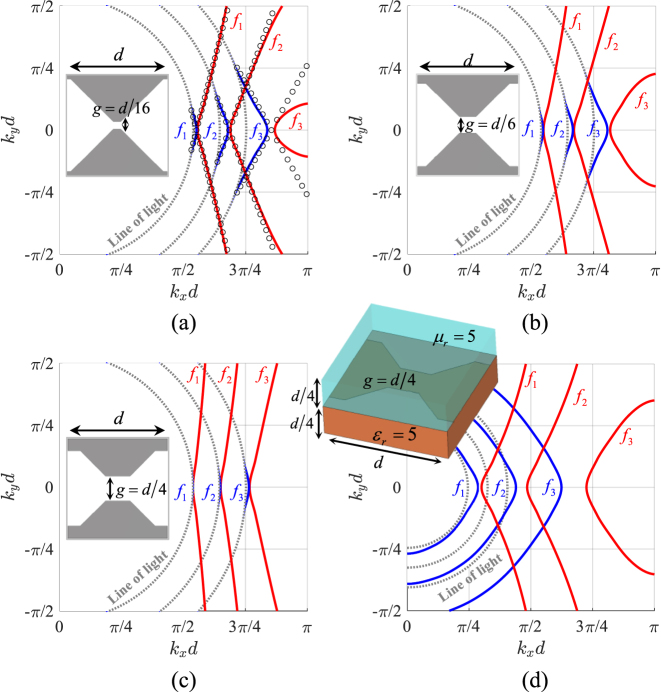
Comparison of the IDCs for SC-MTS at frequencies *f*_1_ = 20 GHz, *f*_2_ = 24 GHz, *f*_3_ = 28 GHz for different geometries; (a)–(c) checkerboard-type MTS with different values of the gap *g*. (d) Geometry as in (c) with a bottom substrate with relative dielectric permittivity *ϵ*_r_ = 5 and a top substrate with relative permeability *μ*_r_ = 5. The results are obtained using CST Eigenmode Solver. The lattice period is *d* = 4 mm. TM mode: blue line; TE mode: red line. The dots in (a) are obtained from the equivalent circuit model with the following parameters *C*_*x*_ = 73.652 fF, *L*_*x*_ = 0.253 nH, *Cy* = 7.112 fF, *Ly* = 2.617 nH.

Figure 7 compares the IDCs for different SC-MTS geometries with the same lattice period *d* = 4 mm. Figure 7(a)–(c) correspond to checkerboard-type structures with different gaps (see insets of the various figures). It is seen that, for a given frequency, the slope of the IDC’s asymptotes increases gradually when the gap increases, due to the decrease of the *x*-directed inductance and the *y*-directed capacitance. It is also seen that the direction of the group velocity is quite stable over a large range of frequencies. The components of the equivalent circuits in Figure 3(b) and (c) have been derived from the dispersion diagram along the *x* axis using an efficient rational function fitting methodology, called VECTFIT [49], and are *C*_*x*_ = 73.652 fF, *L*_*x*_ = 0.253 nH, *Cy* = 7.112 fF, *Ly* = 2.617 nH. The dots in Figure 7(a) are obtained from the analysis of the resulting equivalent circuit. Figure 7(d) shows the deformation of the IDCs when the SC-MTS is loaded with a 1 mm thick substrate of relative permittivity *ϵ*_r_ = 5 and a 1 mm thick superstrate of relative permeability *μ*_r_ = 5. The effect of this loading is to decrease the slope of the asymptotes; the structure is not anymore self-dual and therefore the phase velocities of TM and TE wave along the *x* axis differ each other, as expected. We note that in this case the *q*-TM mode exists for all the propagation angles. This suggests that loading materials with tunable dielectric and magnetic properties can be used to independently control the iperbolicity of the two orthogonal modes, and therefore their focusing directions when excited by a point source.

## 4 Degeneracy of the dual-mode IDCs into straight lines

### 4.1 Single layer structure

**Figure 8:**
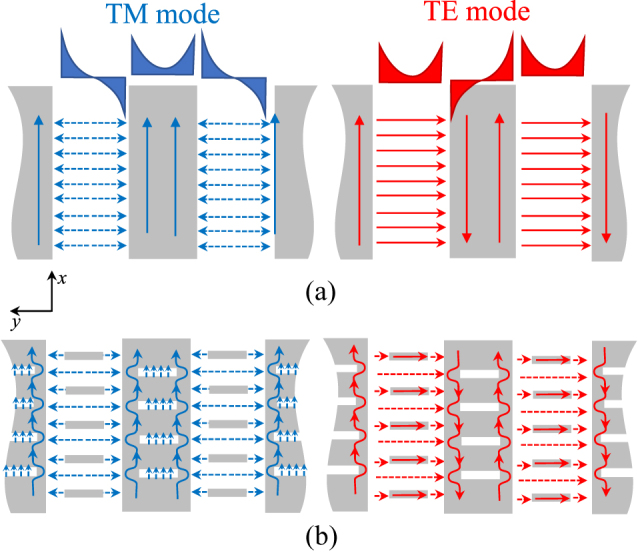
Schematic distribution of currents and fields in a self-complementary strip grid without (a) and with (b) dipole/slot loading. Blue lines: TM mode; red lines: TE modes. Dashed line: electric-field, continuous line: electric currents. The dipoles affect mainly the TE mode while the slots affect mainly the TM mode.

Degeneracy of IDCs into straight lines tangential to the visible circles (Figure 5(c)) is foreseen by the adopted model when $\omega_{B}/\omega_{0}=\sqrt{C_{x}/C_{y}}=\sqrt{L_{y}/L_{x}}\gg 1$ , namely when one reduces the inductance along *x* and the capacitance along *y*, thus leading to the super canalization property described in Figure 5(c). The simplest example in which this happens is a grid of PEC strips directed along the *x*-direction in free space, with strip width equal to half a period (Figure 8(a) and (b)). An electric (magnetic) vertical dipole placed on that structure excites a TM (TE) superfocused mode (see Figure 5(c)). The sketch of electric currents and fields of the two modes is shown in Figure 8(a) (absence of phasing along *y* is assumed). The TM mode exhibits an even symmetry with respect to the center of strip, and an odd symmetry with respect to the center of the slot: on each PEC strip, the currents go in the same direction at the two edges, while the electric field has a null at the center. For the TE mode, the currents change sign  from one edge to the other of the same strip and have a null at the strip center, while the electric field in the slots has always the same direction.

**Figure 9:**
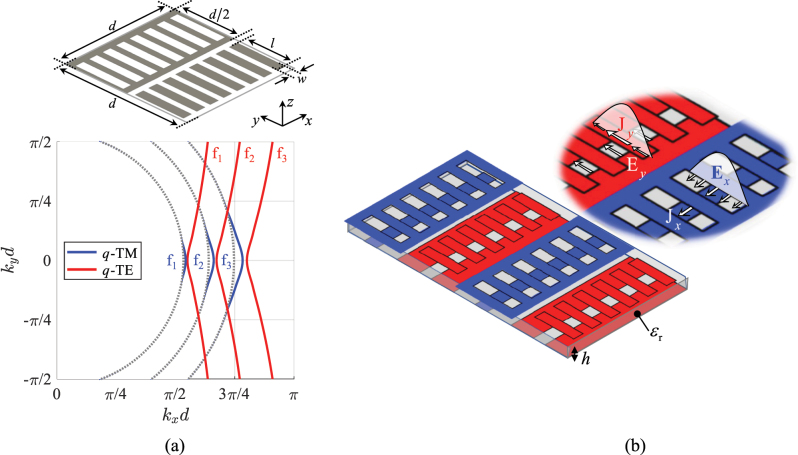
Single layer and two layers dipole-slot structures. (a) IDCs of the dipole-slot single layer geometry at frequencies *f*_1_ = 20 GHz, *f*_2_ = 24 GHz, *f*_3_ = 28 GHz. The dimensions are *d* = 4 mm, *l* = 1.6 mm and *w* = 0.3 mm. (b) Two layers global structure containing slots coupled to strips (metal in blue) and dipoles coupled to slits (metal in red). On top: the zoomed view shows the distribution of electric currents and electric field for both *q*-TE (*E*_*y*_, *J*_*y*_) and *q*-TM (*E*_*x*_, *J*_*x*_) modes propagating along *x*.

This structure has straight IDC lines; however, it is very difficult to use in practice for all those applications that require impedance modulation, due to the lack of tunable geometrical parameters. In fact, the effective propagation constant along the *x*-direction is always very close to the one of free space. The phase velocity along *x* may be decreased by adding *y*-directed dipoles in the empty space and etching *y*-directed slots in the metal part, while maintaining the self-complementarity (see Figure 8(b)). For the TM mode, both the inductance and the capacitance along the *x*-direction will be enhanced by the presence of slots and dipoles, respectively; for the TE mode, both the *y*-directed capacitance and inductance increase. Eventually, both *ω*_*B*_ and *ω*_0_ decrease, but the ratio *ω*_*B*_/*ω*_0_ decreases, thus, reducing the angle Ψ defining the slope of the asymptotes. The IDCs for this structure are shown in Figure 9(a).

Finally, it is concluded that with this single-layer structure it is not possible to control the phase and group velocity and modulate the equivalent impedance simultaneously and independently for the two modes. A better control can be achieved by a dual layer structure, as described in the following section.

### 4.2 Coupling complementary layers

Interesting effects occur when two self-complementary layers are coupled each other in such a way that the dipole part of one layer goes on top of the slot part of the other layer and vice-versa. An example is shown in Figure 9. There, a slot-dipole structure like the one in Figure 8 is tightly coupled to a layer of slits and strips along the *x*-direction. The two surfaces are coupled so that the slots are on top of a strip and the dipoles are on top of a slit. This generates an increase of capacitance along *y* and a decrease of the capacitance along *x*, thus increasing the ratio $\omega_{B}/\omega_{0}=\sqrt{C_{y}/C_{x}}$ , while maintaining the phase velocity lower than the speed of light in the *x*-direction. The IDCs of the relevant *q*-TE and *q*-TM modes are almost vertical straight-lines, as shown in Figure 9(a). However, since the structure is not constituted by an infinitesimal layer, it does not rigorously respect the Babinet’s principle, and therefore the dimensions of dipoles and slots should be adjusted to obtain the mode degeneracy, especially for larger separation distances.

**Figure 10:**
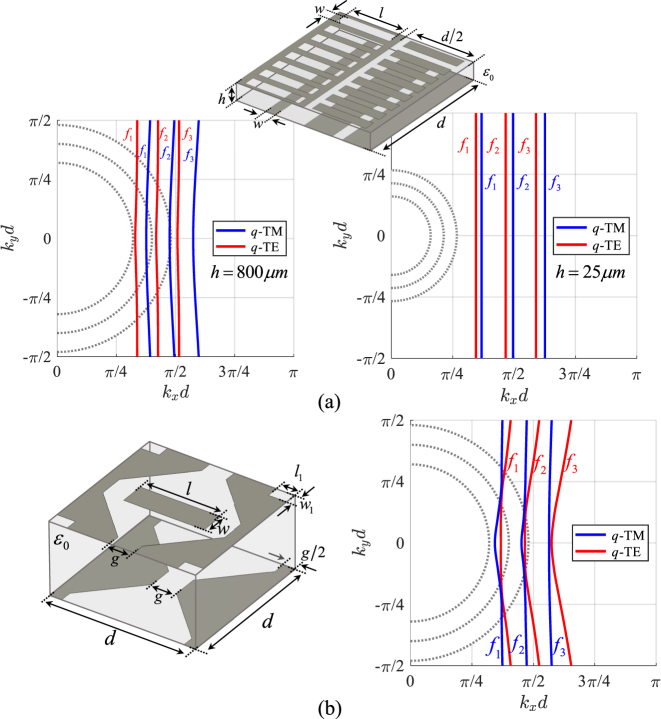
Dispersion analysis of two different two layers dipole-slot structures. (a) Dipole-slot loaded strips coupled to a strip-slit MTS: IDCs at frequencies *f*_1_ = 12 GHz, *f*_2_ = 15 GHz, *f*_3_ = 18 GHz for *h* = 0.81 mm and *f*_1_ = 6 GHz, *f*_2_ = 8 GHz, *f*_3_ = 10 GHz for *h* = 0.025 mm. The geometrical parameters are: *d* = 4 mm, *l* = 1.6 mm, *w* = 0.3 mm. (b) Dipole-slot loaded checkerboard coupled to a strip-slit MTS: IDCs at frequencies *f*_1_ = 12 GHz, *f*_2_ = 15 GHz, *f*_3_ = 18 GHz. The geometrical parameters are *h* = 0.81 mm, *g* = 0.67 mm, *l* = 3.25 mm, *w* = 0.67 mm, *l*_1_ = 0.5 mm and *w*_1_ = 0.335 mm.

A higher design flexibility is obtained by using coupled complementary layers like the ones in Figure 10(b), where the upper layer consists of a checkerboard-like MTS loaded by dipoles and slots. This structure exhibits advantages with respect to the one in Figure 8 because of the presence of more degrees of freedom to control the structure. We note that in both cases of Figures 8 and 10 the superfocusing maybe generated in a large frequency band.

## 5 Dual polarized leaky-wave antennas

**Figure 11:**
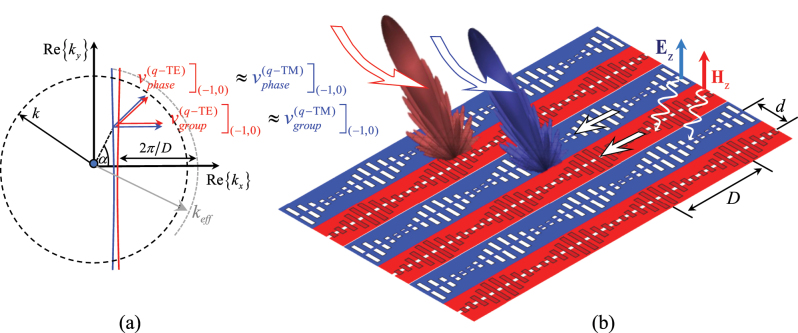
Modulated self-complementary MTS realized through printed subwavelength modulated dipoles and slots fed by strips and slits on the bottom. An impressed phasing *kv*_0_ is applied across the channels. (a) Isofrequency dispersion curves for *q*-TE and *q*-TM surface waves and (b) for the (*n*, *m*) = (−1, 0) indexed modes (leaky waves), obtained shifting to the left of a distance of 2*π*/*D* the IDCs for the zero mode, where *D* is the period of the modulation along *x*. The direction of the energy (group velocity) is always along the strip even when a transverse phasing is applied.

The structures in Figure 10 can be used to design dual polarized leaky wave antennas (Figure 11). Indeed, by modulating the impedances of the complementary strips with a period *D*, one can generate a discrete dual-mode Floquet-wave series with transverse wavenumbers (*k*_*xn*_, *k*_*ym*_) centered around the TM and TE SW-modes. The isofrequency dispersion curves associated with the (*n*, *m*) = (−1, 0) modes are therefore approximately obtained by translating the ones of the (0,0) modes of the quantity −2*π*/*D* in the *k*_*x*_ direction. We notice that in this translation the group velocity remains directed along the *x*-direction, which actually means that the individual strips can be seen as parallel channels that do not couple each other, irrespective of the transverse phasing. The (−1,0) indexed mode radiates in a direction that forms an angle $\arccos\left[\left(k_{\text{eff}}-2\pi /D\right)/k\right]$ with the direction of the strips and an angle arccos(*v*_0_) with respect to *y*, where *kv*_0_ is the phase gradient of the channels in the *y*-direction. It is worth noting that the IDC of the (−1,0) mode maps directly into the embedded element patterns of one channel of the MTS array; therefore, there is the possibility to scan a beam into an LW phased array along straight vertical lines of the *u*–*v* plane with a low coupling and with a dual polarization. The main difficulties in designing leaky wave antennas based on this concept are the following:

– A ground plane is necessary to prevent back radiation.
– The leakage constant is not the same for the two modes.

The two points above are inherently related each other. The ground plane does not alter the zero-indexed mode of the structure if it is sufficiently far from the dual-layer SC-MTS, since the TE-TM SW modes are attenuated along the direction normal to the structure. However, the −1-indexed mode is influenced by the ground plane, and this may lead to different leakage constants for the two modes. Furthermore, additional modes are supported by the structure in presence of the ground plane, and care must be taken not to excite these modes. However, the two aforementioned difficulties can be overcome with a proper design, thus, obtaining the same performance for the beams in the two polarizations. Overall, this structure has many advantages with respect to other leaky wave antennas:

– The channels do not couple each other, even in absence of barriers between contiguous channels and even if the channels width is very small in terms of the operative wavelength.
– The beams of the two polarizations can be controlled independently through the transverse feeding.
– The presence of a tunable material in contact with the two-coupled MTS layers can provide a tuning of *k*_eff_, and therefore a scanning in the vertical direction.

A prototype dual polarized leaky-wave antenna based on the concept previously described has been fabricated and tested. The structure is similar to the one in Figure 11, with dimensions properly scaled to operate around 2.6 GHz. A dog-bone element is added for each period to slots and dipoles to equalize the dispersion of the TE and TM curves. A ground plane is also introduced underneath, together with parasitic dipoles on a superstrate placed on top of the dog-bone dipole. The distance between cross-polarized channels is a quarter of wavelength at 2.6 GHz. The design methodology for the final optimized structure and the details of the feeding are presented in a separate work [50], and are outside the scope of this paper.

**Figure 12:**
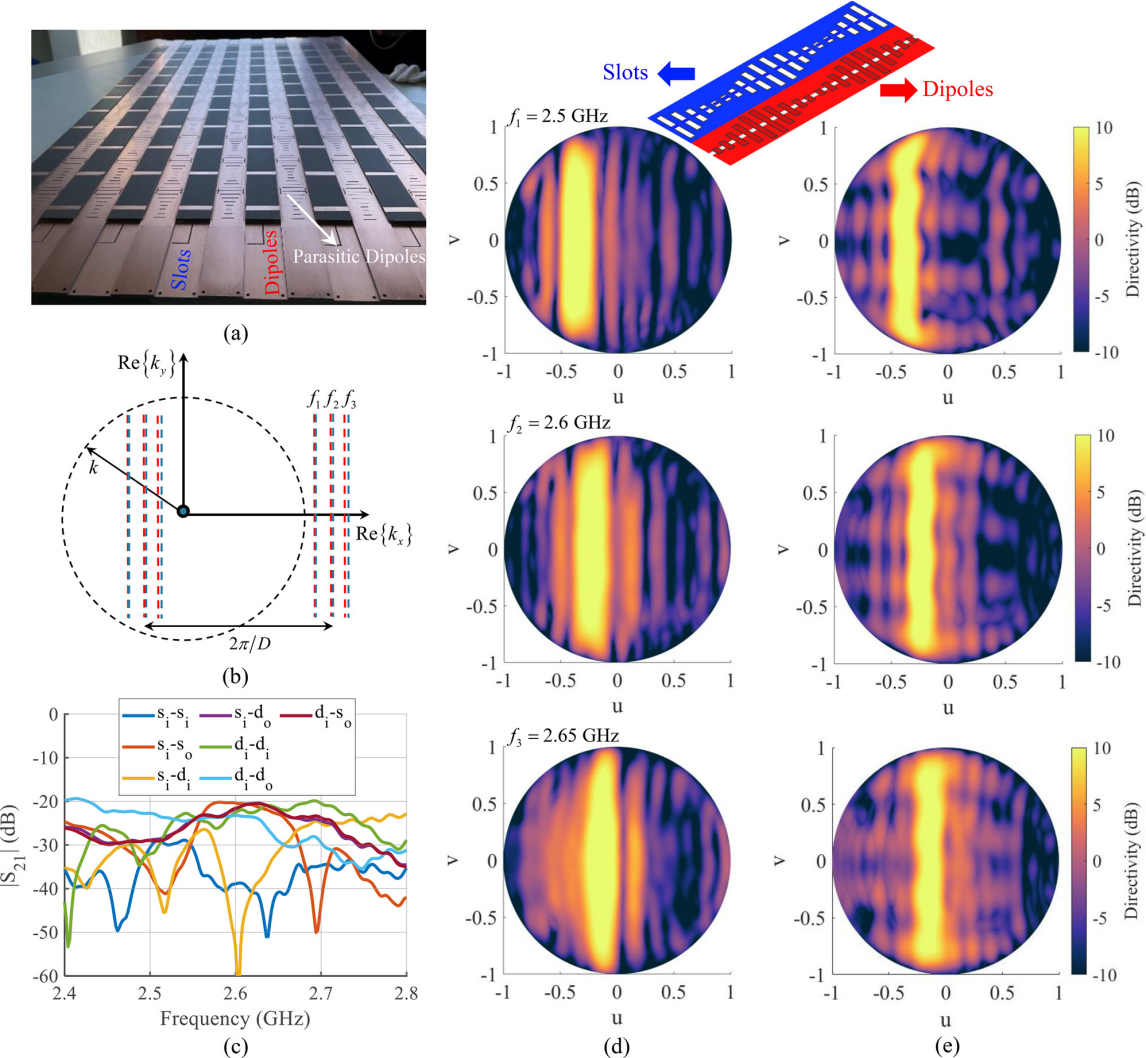
Experimental validation of the dual polarized leaky-wave antenna. (a) Picture of the prototype antenna. (b) Illustration of the isofrequency dispersion curves for the infinite periodically modulated structure. (c) Measured coupling coefficients at the ports of the central channels (“s” and “d” indicate slot and dipole channels, respectively, “i” and “o” are referred to input and output ports, with reference to the antenna sides); (d) and (e) measured embedded element patterns at different frequencies *f*_1_, *f*_2_ and *f*_3_ for slot and dipole channels, respectively.

Here, we focus on the embedded element pattern obtained at different frequencies and on the coupling parameters between ports on the same side and opposite sides of the antenna. A picture of the antenna is presented in Figure 12(a). Figure 12(b) illustrates the straight-line dispersion diagrams obtained by the infinite periodic structure, and their spectral shift of a quantity 2*π*/*D*, where *D* is the modulation period. Figure 12(c) shows the absolute value of the coupling coefficients *S*_12_ as a function of frequency for several couples of ports in the antenna. In the legend inside Figure 12(c), the subscripts “i” and “o” denote input and output ports, respectively, namely ports located on opposite sides of the antenna. The letters “d” and “s” denote instead the channels of “dipole” and “slot” type, respectively. The measured channels are chosen at the center of the antenna. When the letter “d” or “s” are repeated in the legend, like for instance in “*d*_*i*_–*d*_*i*_”, the |*S*_12_| corresponds to contiguous co-polarized channels, namely to channels at a distance half wavelength each-other. As it is apparent from the results of Figure 12(c), all the channels exhibit at any ports a coupling coefficient smaller than −20 dB throughout the inspected frequency bandwidth. These results clearly demonstrate that all the ports are significantly decoupled, despite the fact there is no physical separations between the channels. The series of vertical pictures in Figure 12(d) and (e) represent the embedded element far-field pattern in the u-v plane of slot and dipole channels, respectively, at three different frequencies. For both TE and TM polarizations, the embedded element pattern is concentrated around constant values *u* = *u*_1_, *u* = *u*_2_, *u* = *u*_3_ at the three frequencies *f*_1_, *f*_2_, *f*_3_ (with backward radiation) reported in the figure. This demonstrates that the IDCs are straight lines (as from Figure 12(b)). In [50], it is also shown that this embedded element patterns allow one to synthesize orthogonal beams in any direction along *u* = *u*_*n*_ by properly phasing the channels.

## 6 Summary and extension to optical wavelengths

This paper has investigated the dispersion properties of a particular class of SC-MTS. It has been shown that single layer structures can simultaneously support TE- and TM-polarized SWs, degenerate along one principal direction, and exhibiting hyperbolic IDCs. This behaviour has been explained through the analysis of a simple equivalent circuit, showing how the hyperbolas degenerate into straight lines tangent to the visible region circle for certain values of the equivalent circuit components. These straight IDCs correspond to an SW canalization regime, characterized by an extremely flat phase front, that can be exploited to perform imaging and lensing with enhanced resolution. Furthermore, it is shown that by coupling two SC-MTS layers it is possible to also locally control the phase velocity of the SWs, which paves the way to a wider class of applications, based on equivalent impedance modulation. One of these applications is related to the design of leaky wave antennas through the periodic modulation of the SC-MTS. In this case, the proposed structure provides the possibility to independently control two cross-polarized beams with strong decoupling.

Although numerical results and experimental validation are relevant to centimeter waves, the properties discussed in the paper also apply at higher frequencies, up to far infrared frequencies, as long as one can have electrically thin structures with high sheet conductivity, complementary dispersion of the constituent materials and low absorption losses [51, 52]. At infrared or shorter wavelengths metals exhibit strong dispersion and high absorption losses in plasmonic windows, therefore, the proposed metasurfaces can fulfil the Babinet’s principle only in a narrow spectral range in the vicinity of resonances. Another approach to implement the self-complementary for optical frequencies consists in using all-dielectric materials (such as silicon and oxides) exhibiting a weak dispersion and negligibly small absorption in the optical and near-infrared ranges. The all-dielectric metasurfaces can exhibit the sharp collective resonant response caused by Mie resonances of the intercoupled particles. The latter can be tailored in shape and size to enhance TE or TM local resonance, thus having an equivalent complementary homogenized behavior. The main drawback of this approach is that all-dielectric metasurfaces are not ultrathin being just several times smaller than the incident wavelength in the best case; however, this does not presumably change the dominant effects found here.
